# Novel developmental bases for the evolution of hypobranchial muscles in vertebrates

**DOI:** 10.1186/s12915-020-00851-y

**Published:** 2020-09-09

**Authors:** Rie Kusakabe, Shinnosuke Higuchi, Masako Tanaka, Mitsutaka Kadota, Osamu Nishimura, Shigeru Kuratani

**Affiliations:** 1 Laboratory for Evolutionary Morphology, RIKEN Center for Biosystems Dynamics Research (BDR), 2-2-3 Minatojima-minami, Chuo-ku, Kobe, Hyogo 650-0047 Japan; 2 Department of Biology, Graduate School of Science, Kobe University, Kobe, 657-8501 Japan; 3 Department of Molecular Biology and Biochemistry, Graduate School of Biomedical & Health Sciences, Hiroshima University, Hiroshima, 734-8553 Japan; 4 Laboratory for Phyloinformatics, RIKEN Center for Biosystems Dynamics Research (BDR), Kobe, 650-0047 Japan; 5 Evolutionary Morphology Laboratory, RIKEN Cluster for Pioneering Research (CPR), Kobe, 650-0047 Japan

**Keywords:** Hypobranchial muscle, Development, Lamprey, Hagfish, Shark, Skeletal muscle, *Lbx* genes, Evolution, Vertebrates

## Abstract

**Background:**

Vertebrates are characterized by possession of hypobranchial muscles (HBMs). Cyclostomes, or modern jawless vertebrates, possess a rudimentary and superficial HBM lateral to the pharynx, whereas the HBM in jawed vertebrates is internalized and anteroposteriorly specified. Precursor cells of the HBM, marked by expression of *Lbx1*, originate from somites and undergo extensive migration before becoming innervated by the hypoglossal nerve. How the complex form of HBM arose in evolution is relevant to the establishment of the vertebrate body plan, but despite having long been assumed to be similar to that of limb muscles, modification of developmental mechanisms of HBM remains enigmatic.

**Results:**

Here we characterize the expression of *Lbx* genes in lamprey and hagfish (cyclostomes) and catshark (gnathostome; jawed vertebrates). We show that the expression patterns of the single cyclostome *Lbx* homologue, *Lbx-A*, do not resemble the somitic expression of mammalian *Lbx1*. Disruption of *Lbx-A* revealed that *LjLbx-A* is required for the formation of both HBM and body wall muscles, likely due to the insufficient extension of precursor cells rather than to hindered muscle differentiation. Both homologues of *Lbx* in the catshark were expressed in the somitic muscle primordia, unlike in amniotes. During catshark embryogenesis, *Lbx2* is expressed in the caudal HBM as well as in the abdominal rectus muscle, similar to lamprey *Lbx-A*, whereas *Lbx1* marks the rostral HBM and pectoral fin muscle.

**Conclusions:**

We conclude that the vertebrate HBM primarily emerged as a specialized somatic muscle to cover the pharynx, and the anterior internalized HBM of the gnathostomes is likely a novelty added rostral to the cyclostome-like HBM, for which duplication and functionalization of *Lbx* genes would have been a prerequisite.

## Background

Many characteristics of vertebrate body plan are associated with the extremely complex morphology of skeletal muscles, as compared to the simple contractile tissues of invertebrate chordates, such as those found in amphioxus. Of the vertebrate skeletal muscles, hypobranchial muscles (HBMs), including tongue and infrahyoid muscles in jawed vertebrates, have attracted particular attention in evolutionary studies, due to their peculiar developmental processes. Precursor cells of the HBM originate from somites and migrate along an extensive, arch-shaped pathway circumventing the posterior margin of the posteriormost pharyngeal arch, as a cell stream called the hypoglossal cord [1]. HBMs become innervated by the hypoglossal nerve, the twelfth cranial nerve that runs through the so-called head/trunk interface, overlapping with the hypoglossal cord [2].

The extensive migration of the somitic muscle precursors has been best studied in the formation of paired limbs, another feature shared by vertebrates. At the fore- and hindlimb levels, the ventrolateral lip (VLL) of the epithelial dermomyotome yields de-epithelialized, mesenchymal precursor cells for the limb muscles. The limb muscle precursors lose their original segmental configuration, forming a pool of mesenchymal progenitor cells that migrate distally into the limb buds, where they undergo muscle differentiation [3]. These precursor cells are marked by the expression of *Lbx1*, a member of lady bird class of homeobox transcription factor-encoding genes [4, 5]. In *Lbx1*-deficient mice, the limb muscles are lost or severely affected, with tongue muscles reduced in size [5, 6]. However, *Lbx2*, a paralogue of *Lbx1*, has never been proposed to be involved in myogenesis in either tongue or limbs [7–9].

**Fig. 1:**
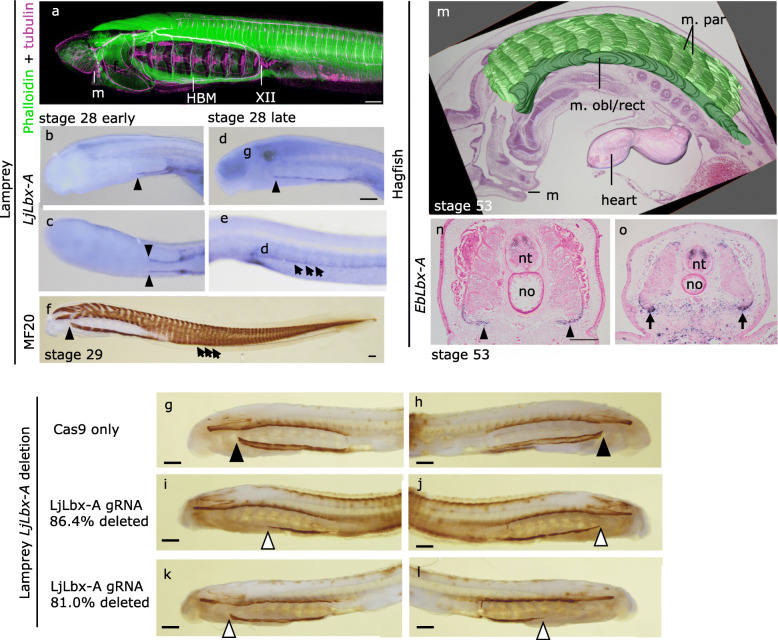
*Lbx-A* gene in expression in lamprey and hagfish embryos. **a** A pre-ammocoete larva doubly stained with phalloidin (green; skeletal muscles) and acetylated tubulin antibody (green; neurons). HBM, hypobranchial muscle; m, mouth; XII, hypoglossal nerve. **b**–**e** Expression of *LjLbx-A* gene in the rostral part of lamprey embryos. Arrowheads indicate the rostral end of hypobranchial muscle (HBM) primordia. Arrows indicate the ventral edge of the extending body wall muscle primordia. **b**, **d** Left lateral views. **c** Ventral view. **e** Left-ventral view. **f** Differentiated somitic muscles visualized by myosin heavy chain antibody MF20. **g**–**l** Anterior portion of the stage 30 embryos stained with MyHC antibody MF20, viewed from the left (**g**, **i**, **k**) and right (**h**, **j**, **l**) sides. Compared to the embryos injected with Cas9 only (**g, h**), *LjLbx-A*-disrupted embryos have thinner and shorter HBM (**i**–**l**). **m** 3D reconstruction of an HE-stained stage 53 hagfish embryo showing location of the hypobranchial-equivalent muscle anlage (dark green; Oisi et al. [17]). Dorsolateral segmented muscles (m. par) are shown by the repeated pattern of light greens. m. obl/rect, anterior oblique muscles and the rectus muscles; m. par, parietal muscles. **n**, **o** Expression of *EbLbx-A* in the trunk of stage 53 hagfish embryo. Arrowheads show the expression at the ventral edge of the somites. Transverse sections at pharyngeal (**n**) and cloacal (**o**) levels. no, notochord; nt, neural tube. Scale bars, 200 mm

In recent developmental studies, the cyclostome lampreys have served as an attractive model that retains the ancestral features of vertebrates [10–14]. One such characteristic is found in the simple morphology of skeletal muscles, e.g., a lack of epaxial/hypaxial distinction of the trunk muscles, as well as the paired fins and associated muscles [15, 16]. On the other hand, lampreys, while lacking the tongue, do possess a putative HBM homologue lying in the superficial layer of the pharyngeal wall, innervated by a nerve called the hypoglossal nerve (XII) in the lamprey (Fig. 1a) [18, 19]. Precursors of the lamprey HBM appear to originate from the ventral side of rostral somites of the late pharyngula (stage 27) [20], first migrating ventrally within the pericardium and then extending anteriorly along the lateral aspect of the pharynx [11, 18, 21–23]. Muscle differentiation of HBMs takes place at the pre-ammocoete larval stage (stage 29; Additional file 1: Fig. S1j), much later than that of other somite-derived muscles. Upon differentiation, HBM myofibers secondarily acquire segmental pattern in conjunction with the pharyngeal arch muscles that derive from unsegmented head mesoderm (Additional file 1: Fig. S1b-f), but not in conjunction with segmentation of the dorsal somitic muscles dorsally overlying the pharyngeal arches (Additional file 1: Fig. S1k and l) [10, 22].

The lampreys, as well as the hagfish, the other group making up the cyclostomes, possess a single cyclostome-specific homologue of the *Lbx* gene, *Lbx-A* (Fig. S2). In the Japanese lamprey *Lethenteron camtschaticum* (formerly *Lethenteron japonicum*), *Lbx-A* (*LjLbx-A*) mRNA has been shown to mark the anteriorly extending HBM precursors [10]. This observation led us to speculate that *LjLbx-A* would retain the ancestral myogenic function of *Lbx* gene in HBM formation acquired early in vertebrate evolution. However, it remains unclear how *Lbx1/Lbx2* genes of gnathostomes are functionally related to the single *Lbx* gene of the lamprey. In this study, we aimed to clarify how the duplicated *Lbx* genes are involved in the morphological complexity of gnathostome HBM by comparing myogenesis in the lamprey, hagfish, and shark.

## Results and discussion

### Characterization and functional analysis of the cyclostome *Lbx-A* genes

In the present study, we first examined the functional importance of lamprey *Lbx-A* gene during development. Since *LjLbx-A* sequence reported previously had lacked the 5′ part of the coding region [10], we isolated a full-length cDNA clone of *LjLbx-A* utilizing the updated genomic data and the gene modeling (Additional file 1: Fig. S2). Newly identified N-terminus of deduced amino acid sequence of *LjLbx-A* contained a 6-amino acid lady bird domain which is shared with *Drosophila lady bird* and mammalian *Lbx2* genes, but not with mammalian *Lbx1* genes, implicating a possible functional correlation of *LjLbx-A* to *Lbx2* genes of jawed vertebrates [24].

We subsequently carried out the detailed analyses of the expression of *LjLbx-A*. In addition to the expression in HBM (Fig. 1b–d), *LjLbx-A* was also expressed in the ventral edge of the postotic trunk somites across the anteroposterior axis, in addition to the HBM precursor cells (Fig. 1e). These *LjLbx-A*-expressing cells in the trunk extend ventrally to form the body wall muscle (stage 29; Additional file 1: Fig. S1j), differentiating much later than the dorsal part of the trunk muscle (Fig. 1f, Additional file 1: Fig. S1g-j). In the later ammcoete larval stage (~ 50 mm body length), *LjLbx-A* was expressed in a cell layer at the dorsal edge of the myotomes, which would later give rise to the muscles in the dorsal median fin (Additional file 1: Fig. S4) [25].

The broad expression of *LjLbx-A* in the ventral side of the trunk does not resemble the somitic expression of mammalian *Lbx1*, which is expressed only in the occipital and limb levels, but not in the flank [4, 26]. To clarify which of the lamprey larval muscles require the function of *LjLbx-A*, we disrupted the *LjLbx-A* locus during embryogenesis, using CRISPR/Cas9-mediated gene editing (Fig. 1g-l, Additional file 1: Fig. S5 to S8). After injection of the Cas9 protein and gRNA complementary to *LjLbx-A*, the dorsal somitic muscles of the larvae appeared unaffected, whereas HBM was lost partly or entirely from the ventral floor of the pharynx (Fig. 1g-l, Additional file 1: Fig. S6a-c). These embryos also lost body wall muscles, suggesting that *LjLbx-A* is required for the formation of both HBM and body wall muscles (Additional file 1: Fig. S6d-f). We also found that *LjLbx-A-*depleted embryos lost the expression of *LjPax3/7-A* in the pharyngeal region, which marks the extending HBM primordium (Additional file 1: Fig. S7) [10, 23], suggesting that the loss of HBM and body wall muscles is attributable to the insufficient extension of precursor cells rather than to hindered muscle differentiation.

We also characterized the *Lbx* gene of the hagfish *Eptatretus burgeri*, *EbLbx-A* (Additional file 1: Fig. S3). In hagfish embryos, *EbLbx-A* was expressed in the ventral edge of the somites (Fig. 1n, o) [17]. This region has been reported to give rise to the anterior oblique muscles (m. decussatus) and the rectus muscles in the ventrolateral aspect of the pharyngeal wall (m. obl/rect; Fig. 1m) [17]. M. obl/rect have been proposed to characterize the HBM of the hagfish, despite the fact that these muscles are innervated by the occipitospinal nerves that pass ventrally as individual segmental nerves, not by a bundled hypoglossal nerve which is the case in the lamprey HBM (Fig. 1a). The morphology of hagfish m. obl/rect also differs significantly from that of the lamprey HBM; the oblique muscles broadly cover the surface of the ventral body from caudal to the mouth to the cloaca, and the rectus muscles lie longitudinally close to the ventral midline. Nevertheless, m. obl/rect have been reported to differentiate at a later stage of embryogenesis [17], which is also the case in the development of the lamprey HBM and body wall muscles (Additional file 1: Fig. S1). The fact that the expression of *EbLbx-A* is restricted to the ventral somites supports the HBM nature of the m. obl/rect, suggesting that the *Lbx*-dependent regulation of differentiation would be conserved in the cyclostomes.

### Catshark *Lbx1* and *Lbx2* are differentially expressed during myogenesis

**Fig. 2:**
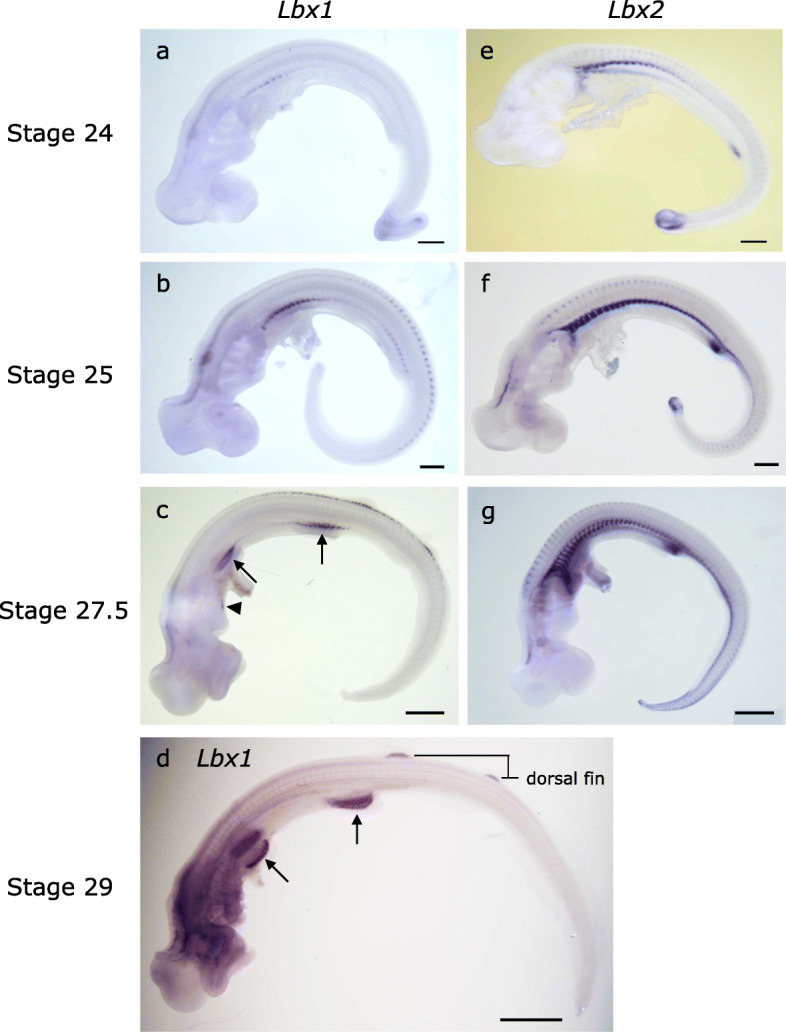
Expression *of Lbx1 and Lbx2* in *S. torazame*. Expression of *Lbx1* (**a**–**d**) and *Lbx2* (**e**–**g**) detected by whole-mount in situ hybridization. Arrowhead and arrows indicate the patch of *Lbx1* expression in CMD and paired fin muscle primordia, respectively. Scale bars, 0.5 mm (**a**, **b**, **e**, **f**), 1 mm (**c**, **g**), and 2 mm (**d**)

We chose the elasmobranch cloudy catshark *Scyliorhinus torazame* as a model to investigate how the wide variety of somite-derived hypaxial muscles, such as those in the limbs, appeared in the vertebrate species diverged early in evolution. It has been reported that, similarly to those of Osteichthyan animals, the *Lbx1* gene of shark and skates are expressed in precursor cells in muscles [27, 28]. However, involvement of *Lbx2* in myogenesis in these animals had remained unexplored, and no insights had been obtained with respect to the differential expression of *Lbx1* and *Lbx2*, which emerged possibly due to the two rounds of vertebrate whole genome duplication (Additional file 1: Fig. S3) [27, 29]. In this study, we examined the expression of *Lbx1* and *Lbx2* during embryogenesis of *S. torazame*, using probes specific to each of the two genes. Unlike in mammals, both *Lbx1* and *Lbx2* were expressed in the somitic muscle primordia, although in a non-overlapping fashion, as detailed below (Fig. 2).

**Fig. 3:**
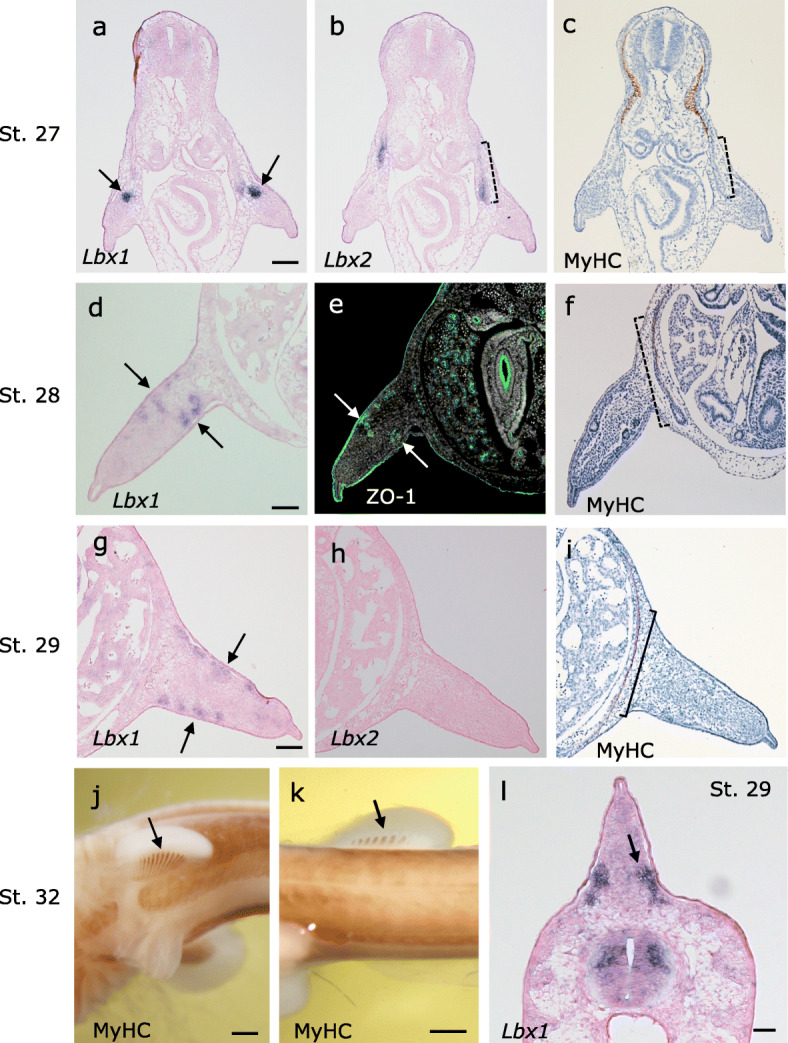
Shark *Lbx1 and Lbx2* are expressed in distinct muscle primordium in the trunk. Transverse sections of *S. torazame* embryos at **a**–**c** stage 27, **d**–**f** stage 28, and **g**–**i** stage 29. **a** Expression of *Lbx1* detected in the pectoral muscle precursor cells (arrows). **b** A serial section to **a**, *Lbx2* is expressed in the ventral somite in the body wall. **c** At the pectoral fin level, *Lbx2* is expressed in rectus abdominus muscle primordia (dotted bracket), which is not yet differentiated, as shown by the absence of MyHC staining (**c**). **d**, **e** *Lbx1* is expressed in the pectoral fin muscle primordia (arrow) which is also stained with ZO-1 tight junction antibody. **g**–**i** At stage 29, *Lbx1* is expressed in the pectral fin muscle primordia which are still not differentiated. Expression of *Lbx2* is no longer detectable. Muscle differentiation is observed only in the rectus abdominus (dotted bracket) but not yet in the fin (**i**). **j**, **k** At stage 32, muscle differentiation is observed in the appendicular (arrow in **j**) and median (arrow in **k**) fins. **l** At stage 29, *Lbx1* is expressed in the muscle precursor cells in the dorsal median fin (arrow). Scale bars, **a**, **d**, **g** 0.1 mm; **j**, **k** 0.5 mm; **l** 0.05 mm

Expression of catshark *Lbx1* commenced at the epithelial VLL of postotic somites and became restricted to the pectoral and pelvic fin levels (Fig. 2a–c). Near the fin buds, these *Lbx1*-positive cells detached from the VLL in bulk, in a similar configuration to the “muscle bud” described by Goodrich (Fig. 3a) [30, 31], to invade the fin bud, where they differentiated into adductor and abductor muscles of the paired fins (Figs. 2a–d and 3d, g, j). Remarkably, these cell aggregates maintained their initial somitomeric pattern and were positive for ZO-1 (zona occuludens-1) antibody, a marker for tight junctions, suggesting that the dermomyotome persisting in the fin muscle primordia is epithelial in nature (Fig. 3e) [32, 33]. This observation is consistent with the classical view that the chondrichthyan fin muscles form from epithelial muscle primordium [30]. *Lbx1* was also expressed in the muscle primordia in median fins (Figs. 2d and 3k, l), similarly to the case in *LjLbx-A* (Additional file 1: Fig. S4).

Catshark *Lbx1* was not expressed in the abdominal muscle primordia (Fig. 3a, g), unlike lamprey/hagfish *Lbx-A*. Remarkably, in contrast to the *Lbx1*, catshark *Lbx2* continued to be expressed in the VLL throughout the trunk (Figs. 2e–g and 3b). *Lbx2*-positive cells did not enter the fin buds; *Lbx2*-positive VLL cells extended ventromedially, passing through the medial aspect of the fin buds (Fig. 3b, f), marking the future abdominal rectus muscle (Fig. 3i). Thus, catshark *Lbx2*, but not *Lbx1*, marks the developing abdominal muscle, a feature similar to that of lamprey *Lbx-A* described above (Fig. 1e).

### Catshark HBM exhibits differential expression of *Lbx1* and *Lbx2* during HBM development

**Fig. 4:**
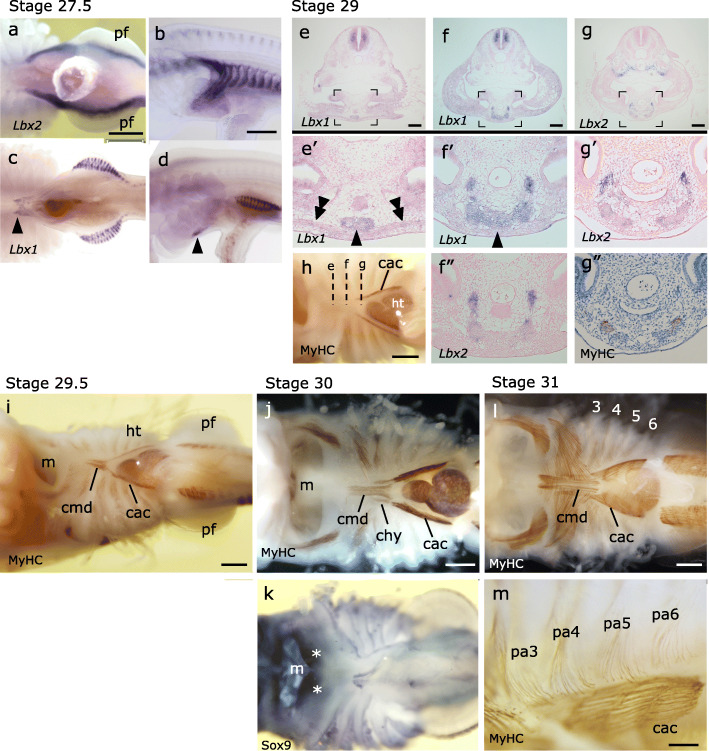
Anterior part of the shark hypobranchial muscles is marked by the expression of *Lbx1*. **a**–**d** Whole-mount in situ hybridization of *S. torazame* embryos at stage 27.5. **a**, **b** Expression of *Lbx2* in ventral (**a**) and left lateral (**b**) views. **c**, **d** Expression of *Lbx1* in ventral (**c**) and left lateral (**d**) views. Arrows show the expression in the anterior extremity of HBM primordium. **e**–**g”** Transverse sections of a stage 29 embryo at the levels indicated in **h** (ventral view stained with MyHC antibody). **e’**–**g’** The enlarged views of the areas marked in **e**–**g**. **f”** and **g”** are the adjacent sections to **f’** and **g’**, respectively, hybridized with different probes. **e’** *Lbx1* is expressed in the medial HBM precursor cells (arrowhead) located dorsal to the hyoid arch-derived muscles (double arrowheads). **f’**, **f”** *Lbx1* is expressed in the medial HBM precursor cells, whereas *Lbx2* expression is restricted in the bilateral HBM primordium (**f”**). **g’**, **g”** Earliest differentiation of HBM occurs in the cells in which *Lbx2* is downregulated. **i** At stage 29.5, the anterior medial part of HBM differentiates as coracomandibularis (cmd) muscle. **j** At stage 30, the anterior tip of early m. coracoarcualis (cac, originally *Lbx2*-positive) extended anteriorly, forming coracohyoideus (chy) muscle connecting cac muscle and basihyal cartilage, overlapping laterally with m. coracomandibularis. Coracomandibularis now forms a single medial bundle of muscle fibers that connects the coracoarcualis muscle and the lower jaw. **k** Meckel’s cartilage expressing Sox9 (asterisk). **l**, **m** The cac muscle has four segments corresponding to the adjacent pharyngeal arches (pa3-6). **l** Ventral view. **m** Left ventrolateral view. ht, heart; m, mouth. Scale bars, **a**–**l** 0.5 mm; **m** 0.2 mm

Our observations also suggested the differential functions of *Lbx1* and *Lbx2* in HBM development in *S. torazame* (Fig. 4). In early embryos, *Lbx2* is expressed in a projection of the cells originating from the anterior somites (Fig. 4a, b, Additional file 1: Fig. S9a). The epithelial VLL of the 3rd and 4th somites released the *Lbx2*-positive cells ventrally along the posterior edge of the pharynx (Additional file 1: Fig. S9a-c). Later, the *Lbx2-*positive cells accumulated adjacent to the heart, then further proceeded anteriorly within the body wall ventral to the pharynx (Fig. 4a, b; Additional file 1: Fig. S9d-f). In contrast, the expression of *Lbx1* was not consecutive along the posterior circumference of the pharynx. At stage 28, a single patch of *Lbx1* expression became evident at the level of the 3rd pharyngeal arch (arrowhead in Figs. 2c and 4c, d, e’). This expression of *Lbx1* in the anteriormost part of HBM primordium did not overlap with the *Lbx2* expression in the midline, whereas dorsally the leading edges of the bilateral portion of HBM transiently express both *Lbx1* and *Lbx2* (Fig. 4f’ and f”). During the extension of HBM primordium from the somites, the anteriormost portion of *Lbx2*-positive domain seems to provide *Lbx1*-positive precursor cells to give rise to the anterior HBM that fuses in the midline.

In late embryos, rows of *Lbx2*-positive cells have differentiated into coracoarcualis (CAC) muscle, the posterior paired domain of HBM (Fig. 4g, g’, h) [34]. CAC myofibers consist of 4 segments each of which corresponding to the posterior pharyngeal arches (Fig. 4l, m; pa3-6), a remarkable similarity to the lamprey HBM (Additional file 1: Fig. S1l). Anatomically, CAC originates at the scapulocoracoid cartilage and inserts to the anterior, medially located HBM (coracomandibularis muscle, CMD; Fig. 4i, j), a morphological orientation consistent with that of the amniote rectus cervicus (sternohyoideus) muscle [11, 34].

*Lbx1*-positive cells, on the other hand, differentiated later than CAC, giving rise to CMD muscle in the midline, located anterior to the CAC muscle (Fig. 4i, j). Unlike the CAC muscle, the CMD muscle was composed of a long single segment of myofibers and inserted to the Meckel’s cartilage (Fig. 4k, l), reminiscent of mammalian geniohyoideus muscle [34].

It is also noteworthy that the entire HBM primordia, including both *Lbx1*- and *Lbx2*-positive cell populations, were stained with the ZO-1 antibody (Additional file 1: Fig. S10). This observation suggests that, in the shark, both the HBM precursor cells and the fin muscle primordia are epithelial in nature, and both remain as a coherent aggregate during the process of extension into the distal parts of the body.

### Duplicated gnathostome *Lbx* genes and complexity of skeletal muscles

**Fig. 5:**
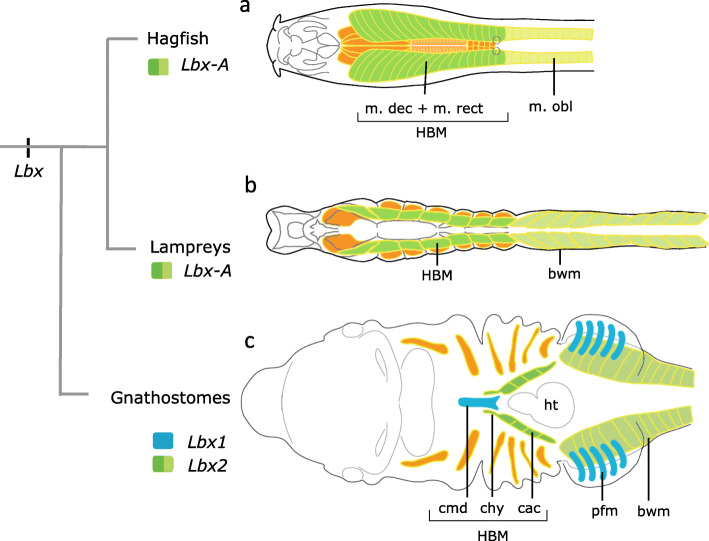
Evolutionary scheme of vertebrate skeletal muscles with reference to the embryonic expression of *Lbx* genes. In the hagfish (**a**), rectus muscle (m. rect) and anterior oblique muscle (m. decussatus) are the hypobranchial muscle counterpart and derived from *Lbx-A*-positive ventral cells of the somites. In the lamprey (**b**), *Lbx-A* is required in the formation of body wall muscles (bwm, light green) and hypobranchial muscle (HBM, dark green). In gnathostomes (**c**), *Lbx1* (blue) and *Lbx2* (green) regulate distinct domains of hypobranchial muscles. *Lbx1* functions in the tongue (cmd, m. coracomandibularis in the shark) and appendicular muscles (pfm, pectoral fin muscles) both of which are lacking in cyclostomes. Pharyngeal muscles derived from head mesoderm are shown in orange

These results provide a new scheme for the developmental homology of HBM elements and appendicular muscles of the vertebrates (Fig. 5). The catshark CAC muscle (associated with *Lbx2* expression) and the lamprey HBM (associated with *Lbx-A* expression) are both attached with the ventral ends of pharyngeal muscles and do not fuse in the midline (Fig. 5b, c) [11]. Moreover, catshark CAC and lamprey HBM are also similar with respect to the pattern of myofiber segmentation which corresponds to the adjacent pharyngeal arches (Additional file 1: Fig. S1k and l; Fig. 4m; Fig. 5b, c), suggesting their muscle differentiation is under the influence of pharyngeal embryonic components such as cephalic neural crest cells and the cranial mesoderm cells. On the other hand, *Lbx1*-positive CMD of the shark, which is homologous to the tetrapod geniohyoideus and genioglossus muscles, seems to be a novel muscular component acquired in gnathostomes (Fig. 5c). CMD-equivalent muscles, as well as the appendicular muscles, are lacking in the cyclostomes, whose HBM is entirely bilateral and segmented in accordance with the pharyngeal arches.

Considering expression patterns of *Lbx1/Lbx2* genes in the wide variety of Osteichthyans, however, the evolutionary pathway seems more complex. In amphibians, only the *Lbx1* gene has been identified in genomic sequences, exhibiting a loss of *Lbx2* locus in the amphibian lineage (Additional file 1: Fig. S3) [8, 29]. In *Xenopus laevis* and direct-developing frog *Eleutherodactylus coqui*, *Lbx1* is expressed in the ventral side of the trunk somites [35, 36]. In *Xenopus*, these *Lbx1*-positive somitic cells give rise to the “rectus abdominus” muscle that extends from the ventral edge of the somites and eventually surrounds the abdomen of tadpoles, similarly to the body wall muscles of other vertebrates. *Xenopus Lbx1* is also expressed in the limb muscle precursors that appear during the metamorphosis [35]. In zebrafish, both *Lbx1b* and *Lbx2* genes were shown to be involved in hypaxial muscles including pectoral fin muscles [8, 37, 38]. It has been suggested that the primary role of *Lbx* transcription factors is to control the switch of proliferation/differentiation of the muscle precursor cells [8, 39]. *Lbx1* and *Lbx2* have been suggested to possess overlapping regulatory functions, as the forced expression of *Lbx2* could rescue *Lbx1* deficiency [8]. Although only limited information about downstream factors of *Lbx* is currently available [40], the comparative insights shown here suggest that skeletal muscles of vertebrate clades have deployed different combinations of *Lbx1* and *Lbx2* to ensure differentiation of complex musculature at variable timing of development.

## Conclusions

Our results indicate that *Lbx1*-positive muscle precursors in the gnathostome ancestor would have been acquired to give rise to the novel, distally located skeletal muscles, i.e., the muscles associated with paired appendages and the anterior internalized tongue (Fig. 5). On the other hand, the posterior portion of the HBM as well as the body wall muscles might represent hypaxial muscle having a deeper evolutionary origin that can be traced back to the common ancestor of vertebrates. Our results lead us to propose a new evolutionary hypothesis—namely, that HBMs, the developmental background of which has long been assumed to be similar to that of limb muscles, may have a closer evolutionary relationship with the abdominal muscle that extensively surrounds the trunk coelom, and that the morphological elaboration of HBMs would have been coupled with the functionalization of duplicated *Lbx* genes.

## Materials and methods

### Obtaining embryos of lamprey, hagfish, and catshark

Mature adults of the Japanese lamprey *L. camtschaticum*, the hagfish *E. burgeri*, and the cloudy catshark *S. torazame* were collected in Hokkaido, Shimane, and Ibaraki prefectures in Japan, respectively. Embryos of each animal were obtained as previously described [41]. Briefly, lamprey eggs were inseminated in vitro and reared at 9°, followed by embryonic staging according to Tahara [20]. The larvae were further cultured in mineral water as described in Higuchi et al. [41] until they reach 50 mm in body length. Hagfish embryos were collected from the sea floor and incubated in artificial sea water, followed by staging according to Dean [42]. Catshark embryos were laid in the fish tank and staged as described previously [43, 44]. Embryos were fixed in 4% paraformaldehyde or Serra’s fixative. The experiments were conducted according to the institutional and national guidelines for animal ethics, approved by the RIKEN Animal Experiments Committee.

### Identification of lamprey and catshark genes

Although we had previously reported partial mRNA sequence of *LjLbx-A* [10], we searched for the full-length sequence with reference to the genomic sequence [45]. A cDNA fragment covering the putative N-terminal Met and the stop codon was obtained. We replaced the previously registered *LjLbx-A* sequence with a new complete cDNA sequence under the same GenBank reference number (HM116241). The mRNA sequence of *E. burgeri Lbx-A* (LC506433) was predicted on the Ensemble Genome Database (transcript ID: ENSEBUT00000001721.1; http://www.ensembl.org) and amplified from embryonic total RNA. We performed a search for genes of the catshark *S. torazame* (*Lbx1*, LC506430; *Lbx2*, LC506431; *Sox9*, LC506432) using the sequence archive Squalomix (https://transcriptome.riken.jp/squalomix/) containing the products of whole genome sequencing [46]. The corresponding cDNAs were amplified from poly-A RNA of stage 24-30 embryos.

### Phylogenetic analysis

Full-length amino acid sequences (summarized in Additional file 2: Table S1) were aligned and phylogenetic trees were constructed by the neighbor-joining method [47] using ClustalX software (http://www.clustal.org) [48]. Positions with gaps were excluded from the analysis. The length of the branches is proportional to the phylogenetic distances estimated using Kimura’s empirical method for protein distances [49]. The scale bar indicates an evolutionary distance of 0.05 amino acid substitution per position in the sequence. The degree of support for internal branches of the tree was assessed in 1000 bootstrap replicates [50].

### Immunohistochemistry

To visualize differentiated muscles and neurons, we used the primary antibodies MF20 or A4.1025 (DSHB, University of Iowa) and anti-acetylated tubulin (T6793; Sigma-Aldrich). ZO-1 (61-7300; Thermo Fisher Scientific) was used to detect tight junctions. The samples were subsequently reacted with the secondary antibody, the F(ab’) fragment of goat anti-mouse IgG (H + L) conjugated with horseradish peroxidase (Thermo Fisher Scientific). Sections were then counterstained with either hematoxylin or DAPI.

### In situ hybridization

Whole-mount and section in situ hybridization of lamprey, hagfish, and catshark embryos was performed as previously described [10, 41]. In brief, paraffin-wax-embedded sections of embryos were deparaffinized, treated with proteinase K, and hybridized with DIG-labeled RNA probe overnight. Hybridized probes were detected with anti-DIG-AP Fab fragments and visualized with reaction with BM purple AP substrate (11442074001; Roche). Sections were counterstained with Nuclear Fast Red (H-2403; Vector Laboratories). The probe sequence of *LjLbx-A* is highlighted in Additional file 1: Fig. S2. The probe template for *EbLbx-A* was amplified with the primers 5′-CGCCCTGGAGGAACTCGCGAG-3′ and 5′-CATTGCCCTTAGCGTAAAGCF-3′. For the catshark, to distinguish the expression of *Lbx1* and *Lbx2*, probes were designed to include the regions highly diverged between the two genes. Primers used to amplify the probe templates were *Lbx1*, 5′-GGACCTGGAGGAGATGAAGG-3′ and 5′-GACATGCGATGCAACAAGG-3′; *Lbx2*, 5′-CGAAGGGACTATTCCTTCTCT–3′ and 5′-AGTCCCACCCCTTCCCCAACAA-3′.

### Knock-down experiment of lamprey *LjLbx-A* gene

Fertilized eggs of the lamprey were microinjected at the 1-cell stage with a mixture of Cas9 protein (0.1 ng/nl, PNA Bio) and guide RNA (gRNA, 25 pg/nl) dissolved in PBS. At appropriate stages, the posterior tail of each embryo was amputated and processed for genomic DNA extraction and PCR amplification for the *LjLbx-A* locus (Additional file 1: Fig. S5). The anterior body part of each embryo was fixed in 4% PFA or Serra’s fixative. After the genomic analyses, individuals with a high deletion rate were examined for phenotypes. As experimental controls, uninjected embryos, embryos injected with Cas9 only, and injected embryos with a low deletion rate were also analyzed and compared (Additional file 1: Fig. S8).

### Library preparation for the screening of CRISPR/Cas9-induced mutants by next-generation sequencing

Each embryo was analyzed for the presence or absence of mutations in the *LjLbx-A* genomic locus by targeted PCR amplification followed by sequencing with Illumina MiSeq. We followed the previously described procedure [51] with modifications. To increase the number of reads and the quality of reads compared to the original protocol, we removed the self-complementary sequence region from one end of the PCR products before the Illumina library preparation (Additional file 1: Fig. S5). The first PCR was performed with 22 cycles using 10 ng of a template DNA extracted from each embryo, with a gene-specific primer containing a 16-nt-long head sequence (head-GS primers; 5′- GCTATGCGCGAGCTGCgtcgagatggactcgctatt-3′ and 5′- GCTATGCGCGAGCTGCgcacgcacgccgggcgacat-3′; Additional file 1: Fig. S2), and the KAPA HiFi HotStart ReadyMix (KAPA Biosystems). Products of each reaction were purified using the AMPureXP beads (Beckman Coulter). The second PCR was performed with 18 cycles, using the full volume of DNA amplified in the first PCR, a head primer containing an 8-nt-long barcode sequence (barcode-head primer; Additional file 3: Table S2), and the KAPA HiFi HotStart ReadyMix. Products of each reaction were purified using AMPureXP beads, quantitated with the Qubit dsDNA HS Assay kit (ThermoFisher Scientific), and combined. Annealing of the GS primer was performed by adding a 200-fold molar amount of the primer to the combined PCR products of 20 ng in a reaction mixture of 24 μl containing 1x ThermoPol Reaction Buffer (NEB), 6 mM MgSO_4_, and 1.4 mM dNTP mix, followed by incubation at 95 °C for 1 min, 60 °C for 1 min, and snap-cooling on ice. Primer extension was performed by adding 1 μl (8 U) of the Bst DNA polymerase (NEB) to the reaction mixture, followed by incubation at 65 °C for 3 min and 80 °C for 20 min, and snap-cooling on ice. The combined PCR product, after the primer extension, was purified using AMPureXP beads, eluted in 10 μl of EB buffer (Qiagen), and further treated with 36 U of S1 nuclease (Takara) in a reaction mixture of 20 μl containing 1x S1 nuclease buffer at 23 °C for 15 min. The reaction was terminated by adding 0.5 μl of 0.5 M EDTA solution, and the DNA was purified using the AMPureXP beads and eluted in 10 μl of buffer EB. The combined PCR product after primer extension and digestion of the single-stranded region was analyzed by TapeStation (Agilent) to confirm the shortened length of the head and the barcode sequences (24 nt) before Illumina library preparation. Library preparation was performed as described previously [52] using 10 ng of the combined PCR product that does not contain the self-complementary region, together with the Illumina TruSeq compatible adapter.

### Amplicon sequencing and detection of deletion on target regions

Sequencing was performed using the library prepared above, without the use of the control PhiX library, on Illumina MiSeq with the MiSeq Reagent Nano Kit v2 (Illumina) to obtain 250-nt-long paired-end reads. Quality control of the sequencing reads was performed using FastQC ver. 0.11.5 (https://www.bioinformatics.babraham.ac.uk/projects/fastqc/). The BBmerge function of BBTools ver. 37.88 [53] was used to merge two overlapping paired reads into a single long read. The merged reads were then trimmed to remove low-quality regions and the Illumina adapter sequences using Trim galore ver. 0.4.2 (https://www.bioinformatics.babraham.ac.uk/projects/trim_galore/). After separating the amplicon reads according to the barcode sequence for each individual (Additional file 3: Table S2), Cutadapt ver. 1.8.3 [54] was used to remove the barcode sequence and the head sequence. The trimmed reads were aligned with the cDNA sequence of LjLbx-A by BBMap of BBTools, and deletion profiles of the CRISPR/Cas9 target sites were visualized and evaluated with the UCSC Integrative Genomics Viewer (IGV) ver. 2.7.2 [55]. The sequencing data have been deposited to the DNA Data Bank of Japan (DDBJ) under the accession number DRA009475.

## Supplementary information

**Additional file 1: Figures. S1-S10.** Skeletal muscle formation during lamprey embryogenesis (Fig. S1). Genomic structure of *LjLbx-A* gene and positions of CRISPR/Cas9 targets (Fig. S2). Phylogenetic analysis of the cyclostome and catshark Lbx genes (Fig. S3). Expression of *LjLbx-A* in the dorsal median fin muscle primordia (Fig. S4). Workflow and additional data for lamprey genome editing experiments (Fig. S5-S8). Expression of catshark *Lbx2* in the extending HBM precursor cells (Fig. S9). ZO-1 staining of HBM primordium in the catshark embryo (Fig. S10).

**Additional file 2: Table S1.** Lbx genes compared in this study.

**Additional file 3: Table S2.** Barcode primers used in lamprey genome editing experiments.

## Data Availability

Newly sequenced genes have been registered in GenBank under the reference numbers shown in the “Materials and methods” section. The amplicon sequencing data of CRISPR/Cas9 experiments have been deposited to the DNA Data Bank of Japan (DDBJ) under the accession number DRA009475 [56]. DNA sequences used in the phylogenetic analyses are listed in Additional file 2: Table S1.
