# Supplementary material for: Novel developmental bases for the evolution of hypobranchial muscles in vertebrates
Source: BMC Biol. 2020 Sep 9;18:120. doi: 10.1186/s12915-020-00851-y (PMC7488077; doi:10.1186/s12915-020-00851-y)
Supplement: Supplementary file 1 — Additional file 1: Figures. S1-S10. Skeletal muscle formation during lamprey embryogenesis (Fig. S1). Genomic structure of LjLbx-A gene and positions of CRISPR/Cas9 targets (Fig. S2). Phylogenetic analysis of the cyclostome and catshark Lbx genes (Fig. S3). Expression of LjLbx-A in the dorsal median fin muscle primordia (Fig. S4). Workflow and additional data for lamprey genome editing experiments (Fig. S5-S8). Expression of catshark Lbx2 in the extending HBM precursor cells (Fig. S9). ZO-1 staining of HBM primordium in the catshark embryo (Fig. S10). [file 12915_2020_851_MOESM1_ESM.pdf]

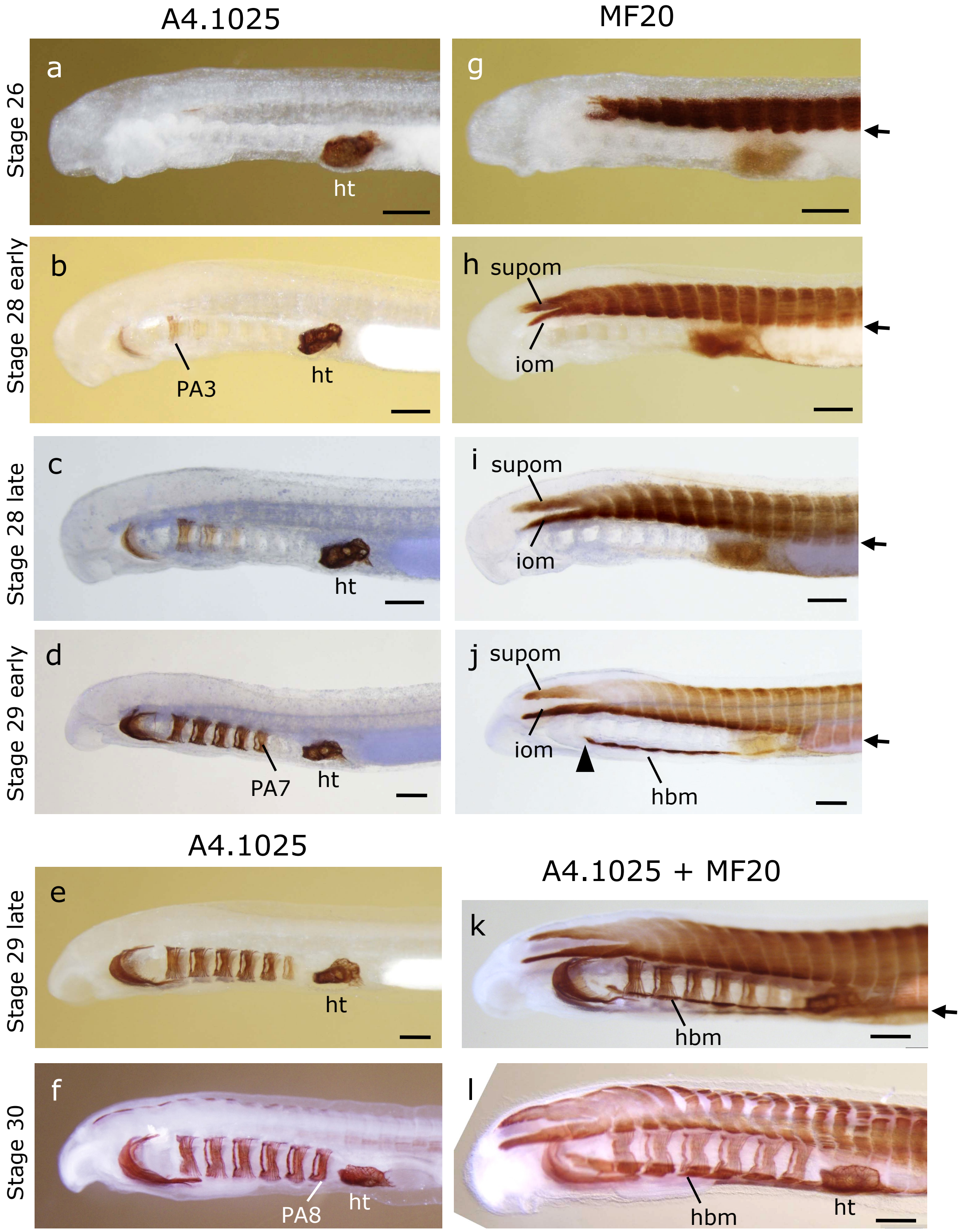

Figure S1. Skeletal muscle formation during lamprey embryogenesis. a-f, Embryonic pharyngeal arch (PA) muscles stained with an MyHC (A4.1025) antibody. Note that differentiation of PA muscles proceeds in anterior-to-posterior order. Cardiac muscles in the heart (ht) is also stained. **g-j**, Somitic skeletal muscles stained with MF20 MyHC antibody. Arrows indicate the ventral edge of the differentiated body wall muscle. Arrowhead in (**j**) indicates the anterior tip of the differentiated hypobranchial muscle (HBM). Developmental pattern of HBM makes a clear contrast to that of other somitic muscles, such as supra- and infraoptic muscles (supom and iom), which are the differentiated myotomes *per se* that have extended anteriorly into preotic region. **k, l,** double staining by A4.1025 and MF20, showing the segmentation of HBM matching the PA muscles. At these stages, the bilateral body wall muscle completely covers the abdominal region and unite at the ventral midline. hbm, hypobranchial muscle; ht, heart; iom, infraoptic muscle; supom, supraoptic muscle. Scale bars, 0.2mm.

a.

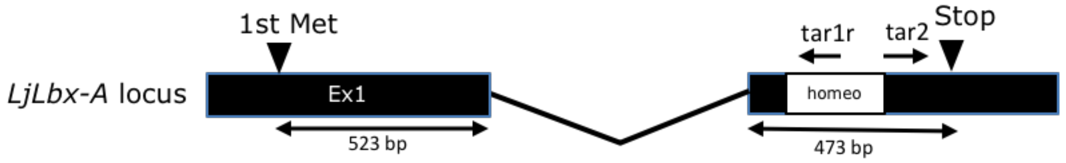

b.

```
         10        20        30        40        50        60        70        80        90
ATCGATGACGACCGCCTTCCAGAAGCCGAAACCCAACCAGGGTCTCCCGCGCGCCATGGAGGAACGGAGCGGGGCCGCTCACCGCTCGA
    M  T  T  A  F  Q  K  P  K  P  N  Q  G  L  P  R  A  M  E  E  R  E  R  G  R  S  P  L  D

        100       110       120       130       140       150       160       170       180
CCACCTCCCGCCGCCGGCCAACTCCAACAAGCCCCTGACTCCCTTCAGCATCGAGGACATCCTCAGCAAGCCATCTGTGCGGCGTCGCGG
 H  L  P  P  P  A  N  S  N  K  P  L  T  P  F  S  I  E  D  I  L  S  K  P  S  V  R  R  R  G

        190       200       210       220       230       240       250       260       270
ATTCGCCGTGTGCGCTGCCAGAGCTTCCGCTGCGGCGGCGGCGGCAGCGGCGGCGGCGCTGGGAGCCGAGGAGGGAGCGGGGCCTAT
 F  A  V  C  A  A  R  A  S  A  A  A  A  A  A  A  A  A  A  L  G  A  E  E  G  A  G  P  M

        280       290       300       310       320       330       340       350       360
GGTTGGGCACGGCGGTGGTGGTGGAGGAGGCGGTGGTGGTGGTGGAGGAGGCGGTGGTGGTGGAGGAGGAGGCGGTGGTGGTGGAGGCGG
 V  G  H  G  G  G  G  G  G  G  G  G  G  G  G  G  G  G  G  G  G  G  G  G  G  G  G  G  G  G

        370       380       390       400       410       420       430       440       450
CGGAGGACACCACGCCGGTGGTCACGGAGCGACCCTAGTCCGACCGTGCACGGTGCAGGCCACGCAGTCGTCCCCGCTGTGCGCGCTGGA
 G  G  H  H  A  G  G  H  G  A  T  L  V  R  P  C  T  V  Q  A  T  Q  S  S  P  L  C  A  L  E
                                                                  Gene-specific primer (forward)
        460       470       480       490       500       510       520       530       540
GGAGTTGGCGAGCAAAACGTTTAAGGGTCTCGAAGTGAGCGTCTTGCAGGCTGCTGAAGGTGCGCTGGGCTTGCCACGTCGAGATGGACT
 E  L  A  S  K  T  F  K  G  L  E  V  S  V  L  Q  A  A  E  G  A  L  G  L  P  R▲R  D  G  L

        550       560       570       580       590       600       610       620       630
CGCTATTTTCGGGCCTCGAACTGCGCCCAAGAAGCGCCGCAAGTCGCGCACGGCCTTCACGAACGCGCAGATCTACGAACTCGAGAAGCG
 A  I  F  G  P  R  T  A  P  K  K  R  R  K  S  R  T  A  F  T  N  A  Q  I  Y  E  L  E  K  R

        640       650       660 tar1r 670       680       690       700       710       720
CTTCCTCTACCAGAAGTACCTCTCGCCGGCCGATCGCGACCAGATCGCCCAGCAGCTGGGCCTCACCAACGCGCAGGTGATCACCTGGTT
 F  L  Y  Q  K  Y  L  S  P  A  D  R  D  Q  I  A  Q  Q  L  G  L  T  N  A  Q  V  I  T  W  F

        730       740       750       760  tar2 770       780       790       800       810
CCAGAACAGGCGCGCCAAGCTCAAGCGGGATCTGGAGGAGATGAAGGCCGACGTGGAGTCGACCAAGAAGATCGACCCGATCGCCCTGGT
 Q  N  R  R  A  K  L  K  R  D  L  E  E  M  K  A  D  V  E  S  T  K  K  I  D  P  I  A  L  V
                                         Gene-specific primer (reverse)
        820       830       840       850       860       870       880       890       900
GGGGGAGGGCGCCCTGCCGACGTCCCTCGTCGAGGCGATGTCGCCCGGCGTGCGTGCCCTCGACAGGAACGACTCACGCGGGGAGACGTG
 G  E  G  A  L  P  T  S  L  V  E  A  M  S  P  G  V  R  A  L  D  R  N  D  S  R  G  E  T  C

        910       920       930       940       950       960       970       980       990
CACCTCGCCCTCCCCGCGCTCCGAGCATAGCCGGGACGACTTCAGCGGCGACGACGACGACGATGACGACGACGACGACATCGAGATCGA
 T  S  P  S  P  R  S  E  H  S  R  D  D  F  S  G  D  D  D  D  D  D  D  D  D  D  I  E  I  D

       1000
CGTGGACGATTAGCGCT
```

Figure S2. a, Genomic structure of *LjLbx-A* locus and target sites for CRISPR/Cas9. **b**, Coding sequence of *LjLbx-A* gene (revised version of GenBank No. HM116241) and positions of CRISPR/Cas9 targets, along with the deduced amino acid sequence. The lady bird domain, conserved in *Drosphila lady bird* genes and mammalian *Lbx2* genes, is highlighted in yellow. The homeodomain is shown in red letters representing amino acids. The nucleotide sequence used for the RNA probe in expression analyses is highlighted in green. Two target sites for gRNA (tar1r and tars) are framed. An intron position is indicated by the triangle. Locations of the primers used in the amplicon sequencing are indicated by the red arrows.

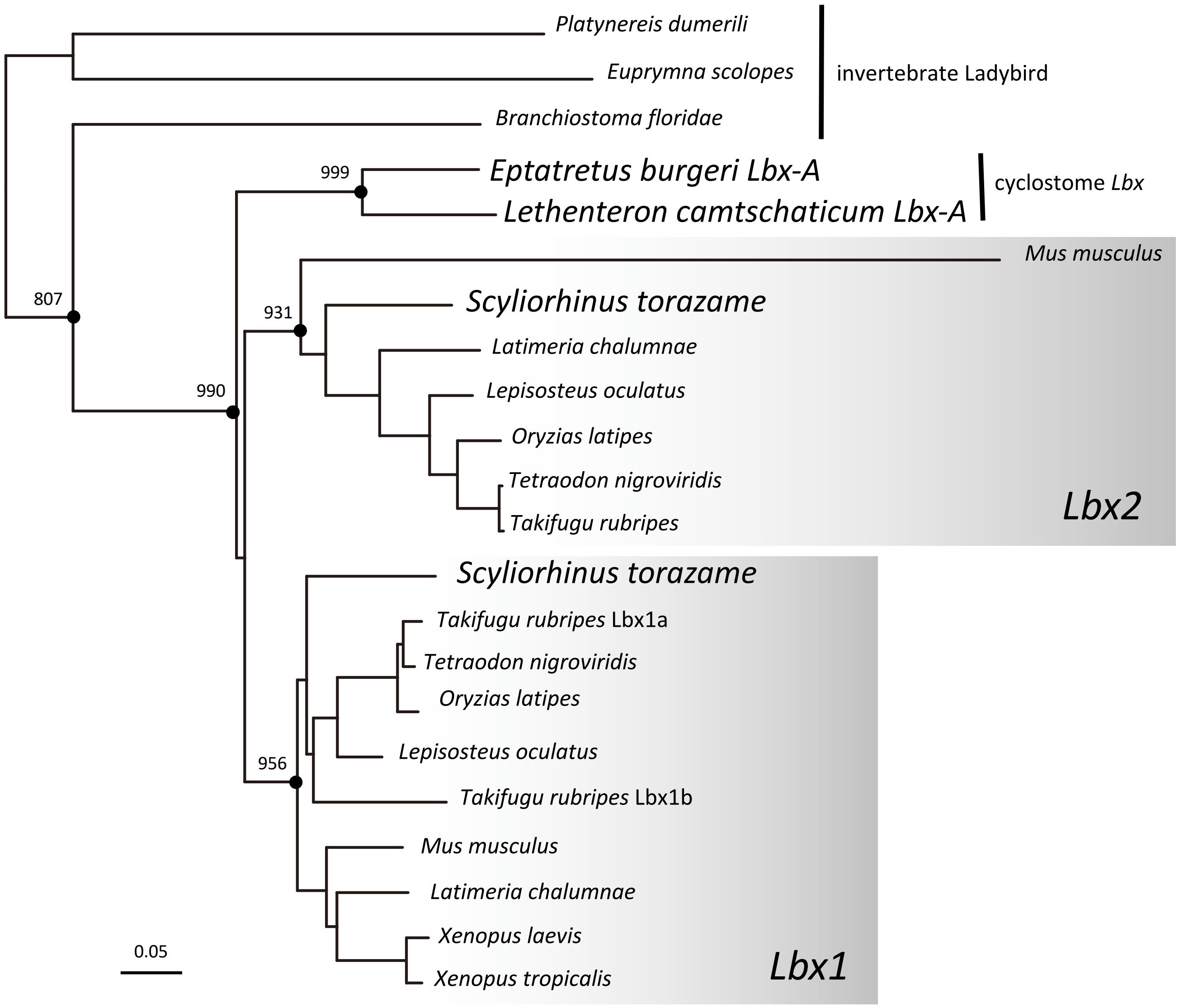

Figure. S3. Phylogenetic analysis of the cyclostome and catshark *Lbx* genes . The phylogenetic tree was constructed with the neighbor-joining method. Length of the branches is proportional to the phylogenetic distances estimated using Kimura's empirical method for protein distances. The scale bar indicates an evolutionary distance of 0.05 amino acid substitution per position in the sequence. The degree of support for internal branches of the tree was assessed in 1,000 bootstrap replicates. References for the nucleotide sequences used in this analysis is shown in Additional Data File 1: Table S1.

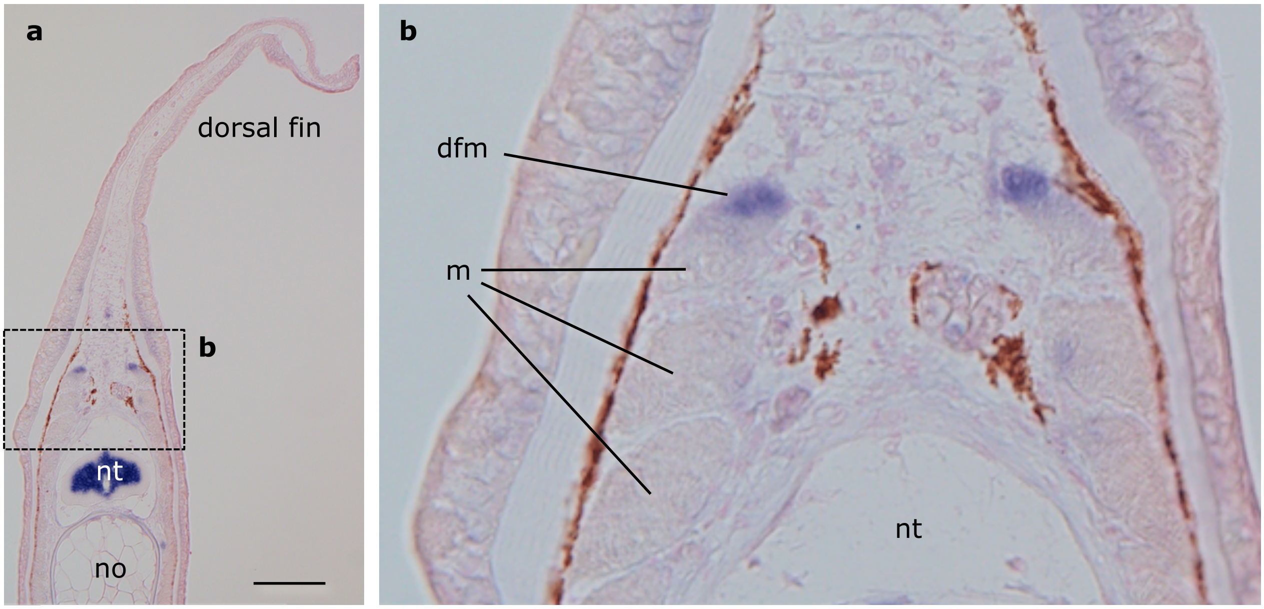

Figure S4. *LjLbx-A* is expressed in muscle primordia of the dorsal median fin in ammocoete larvae. a, Expression of *LjLbx-A* in the transverse section at the pre-anal level of 53-mm ammocoete larva. **b,** Magnified view of the framed area in **a**. *LjLbx-A* expression is specifically expressed in the putative precursor cells of dorsal fin muscle (dfm). m, myotomes; no, notochord; nt, neural tube. Scale bar; 0.1mm.

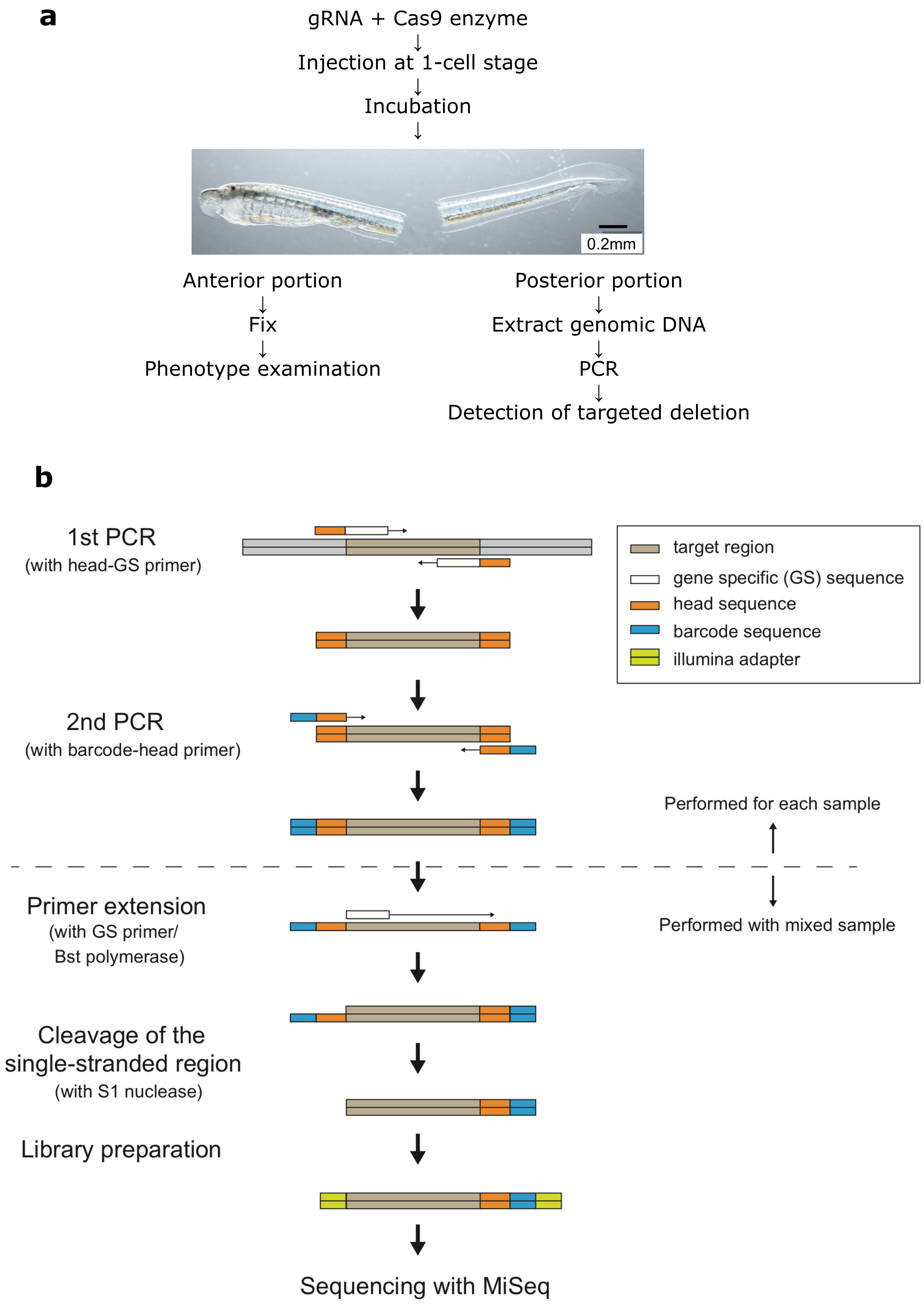

Figure S5. Genome editing experiment on lamprey embryos. a. The workflow of gene editing in this study. **b.** Overview of the amplicon library preparation. Sequences of barcode-head primers are listed in Additional file 3: Table 2.

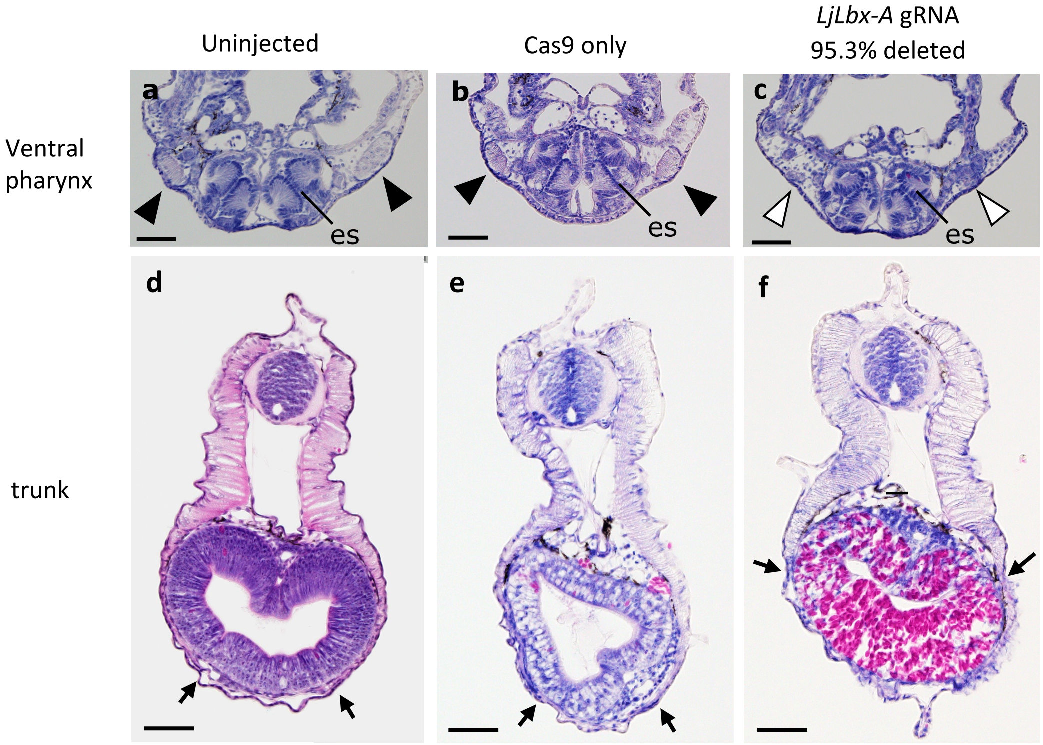

Figure S6. *Lbx-A* gene is required for formation of hypobranchial muscle and body wall muscles in the lamprey. a-f, Phenotypes of *Lbx-A*-deleted lamprey embryos. Results of amplicon sequencing of each embryo are shown in Additional file 1: Fig. S8. Transverse sections at pharynx (**a-c**) and trunk (**d-f**) levels of stage 30 embryos were stained with hematoxilin and eosin. Arrowheads indicate presence (black) and absence (white) of HBM. Arrows indicate the ventral edge of the body wall muscles. In the *LjLbx-A* gRNA-injected embryo, HBMs were absent at the lateral side of the endostyle (es). Ventral edges of the myotomes failed to extend ventrally. For statistical analysis, see Figs. S3 and S4. Scale bars; **a-f**, 0.05mm. For analysis of genomic deletion efficiency, see Additional file1: Fig. S8.

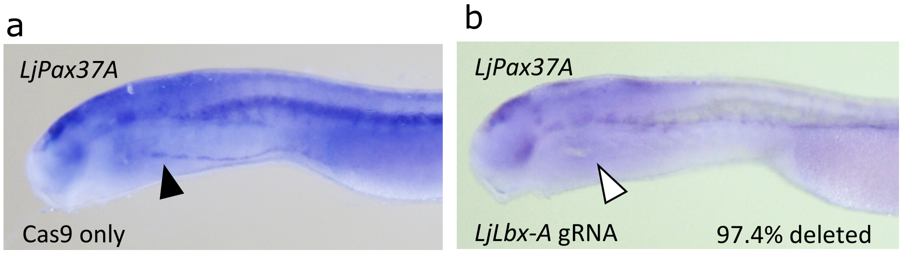

Figure S7. Extension of HBM primordium is lost in *Lbx-A*-depleted embryos. Anterior part of the lamprey embryos injected with cas9 only (**a**) and gRNA/Cas9 (**b**), stained with antisense probe for *LjPax37-A* gene. Arrowheads indicate the leading edge of the HBM primordium (**a**) which is absent in gRNA-injected embryo (**b,** white arrowhead) at stage 28. For analysis of genomic deletion efficiency, see Additional file1: Fig. S8.

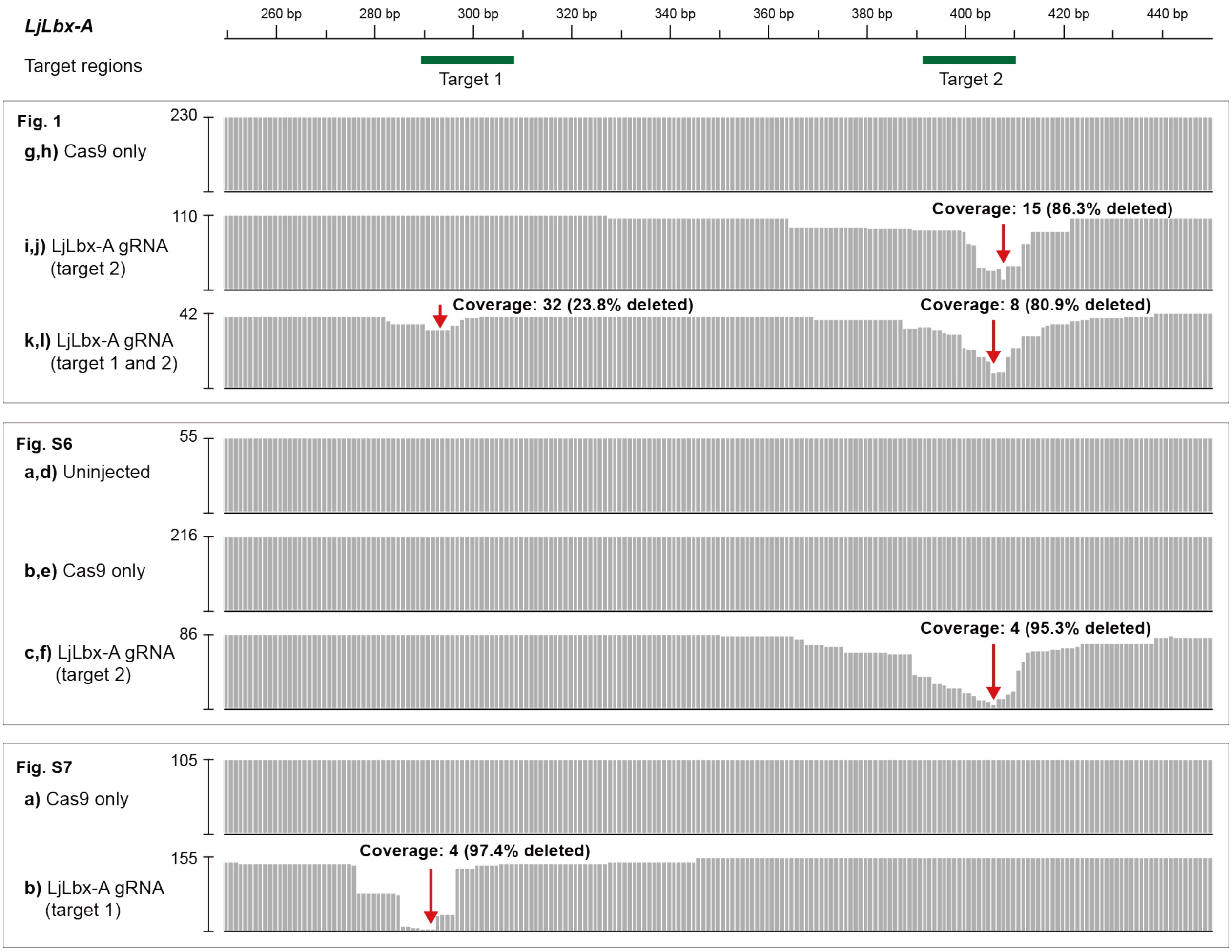

Figure S8. Specific deletion of *LjLbx-A* locus in gRNA-injected embryos. Enlarged coverage view of target sites for *LjLbx-A* CRISPR/Cas9**.** The track captions at the left correspond to individuals shown in Fig.1 and Additional file 1: Fig. S6 and S7. Y-axis shows the depth of sequence reads. A deletional mutation with the most affected site pointed by a red arrow. The displayed deletion rate is the ratio of deletion to the highest read coverage around the target sites.

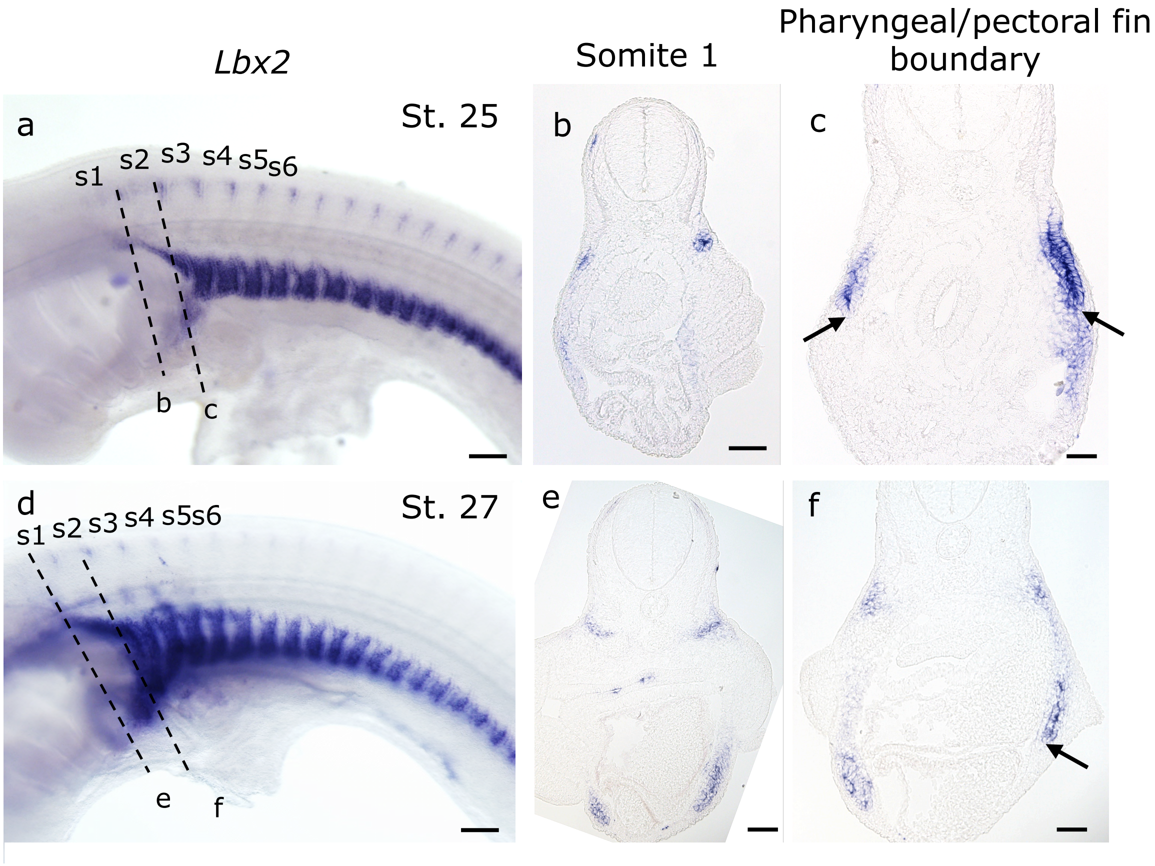

Figure S9. *Lbx2*-positive muscle precursors detach from VLL and accumulated in posterior/ventral region of the pharynx. a and **d**, Expression of *Lbx2* detected by whole-mount in situ hybridization. **b, c, e** and **f**, Transverse sections at the levels indicated in **a** and **d**. Arrows in **c** and **f** indicates VLL. Scale bars; **a** and **d**, 0.2mm; **b**, **e** and **f**, 0.1mm; **c**, 0.05mm.

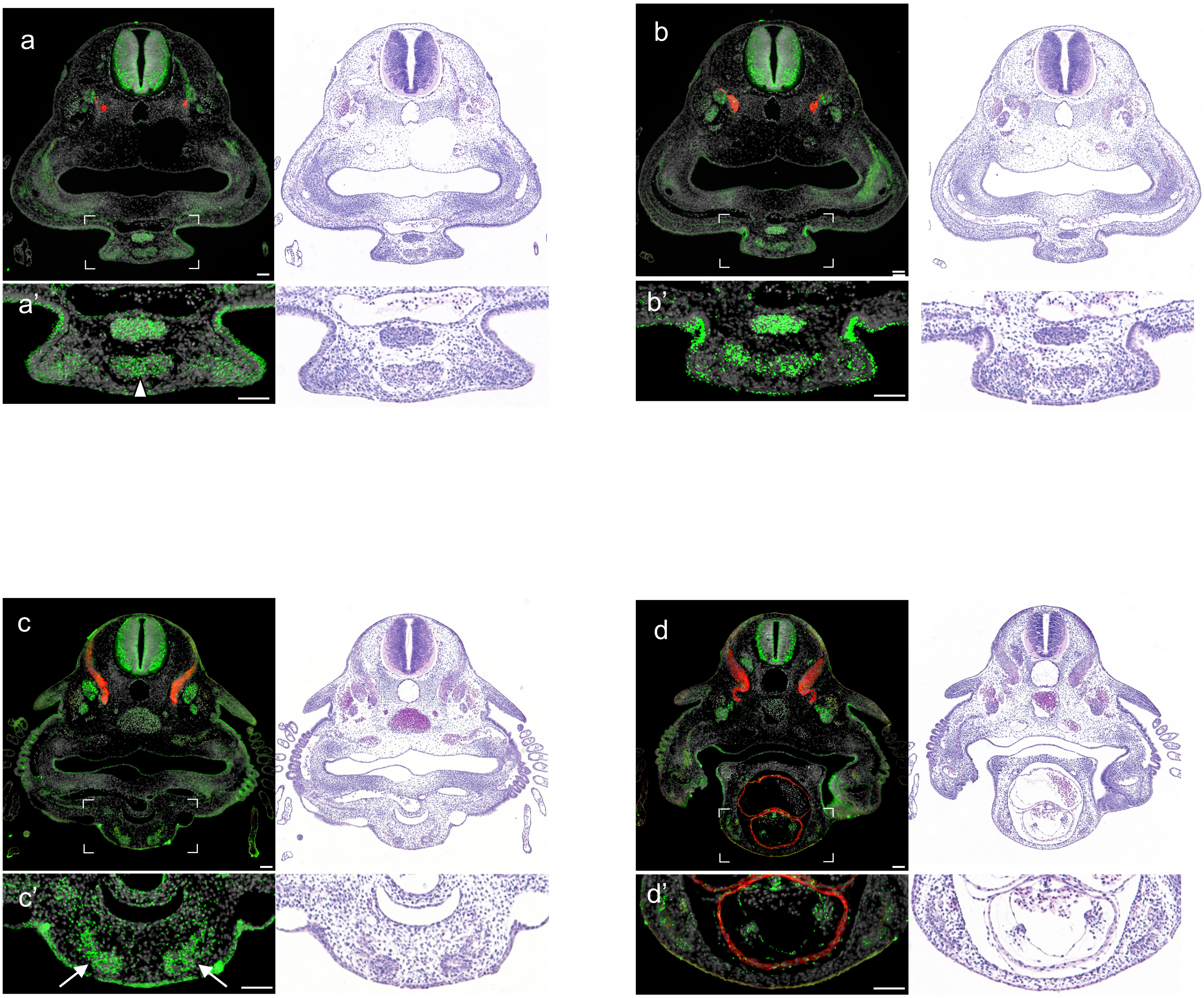

Figure S10. Hypoglossal cord of the catshark is not mesenchymal. Double immunostaining of ZO-1 (green; tight junction) and A4.1025 (red; differentiated myofibers) of a stage 28 embryo, compared with the Hematoxylin-Eosin staining of adjacent sections, at the levels of **a**, anterior to the hyoid arch; **b**, hyoid arch; **c**, rostral esophagus; **d**, heart. **a’- d’** show the enlarged view of the areas marked in **a-d**. Arrowhead (**a’**) indicates the coracomandibularis (CMD) muscle primodium. Arrows (**c’**) indicate the coracoarcualis (CAC) muscle primordia. Scale bars, 0.2mm.
